# Internet-Based, Culturally Sensitive, Problem-Solving Therapy for Turkish Migrants With Depression: Randomized Controlled Trial

**DOI:** 10.2196/jmir.2853

**Published:** 2013-10-11

**Authors:** Burçin Ünlü Ince, Pim Cuijpers, Edith van 't Hof, Wouter van Ballegooijen, Helen Christensen, Heleen Riper

**Affiliations:** ^1^Department of Clinical PsychologyFaculty of Psychology and EducationVU University AmsterdamAmsterdamNetherlands; ^2^EMGO Institute for Health and Care Research (EMGO+)VU University Medical CenterAmsterdamNetherlands; ^3^Division of Online Health Training, Innovation IncubatorLeuphana UniversityLueneburgGermany; ^4^GGZ inGeestRegional Mental Health Service CentreVU University Medical CentreAmsterdamNetherlands; ^5^Black Dog InstituteUniversity of New South WalesSydneyAustralia

**Keywords:** depression, randomized controlled trial, ethnic groups, Internet, psychotherapy

## Abstract

**Background:**

Turkish migrants living in the Netherlands have a high prevalence of depressive disorders, but experience considerable obstacles to accessing professional help. Providing easily accessible Internet treatments may help to overcome these barriers.

**Objective:**

The aim of this study was to evaluate the effectiveness of a culturally sensitive, guided, self-help, problem-solving intervention through the Internet for reducing depressive symptoms in Turkish migrants.

**Methods:**

A two-armed randomized controlled trial was conducted. The primary outcome measure was the severity of depressive symptoms; secondary outcome measures were somatic symptoms, anxiety, quality of life, and satisfaction with the treatment. Participants were assessed online at baseline, posttest (6 weeks after baseline), and 4 months after baseline. Posttest results were analyzed on the intention-to-treat sample. Missing values were estimated by means of multiple imputation. Differences in clinical outcome between groups were analyzed with a *t* test. Cohen’s *d* was used to determine the between-groups effect size at posttreatment and follow-up.

**Results:**

Turkish adults (N=96) with depressive symptoms were randomized to the experimental group (n=49) or to a waitlist control group (n=47). High attrition rates were found among the 96 participants of which 42% (40/96) did not complete the posttest (6 weeks) and 62% (59/96) participants did not complete the follow-up assessment at 4 months. No significant difference between the experimental group and the control group was found for depression at posttest. Recovery occurred significantly more often in the experimental group (33%, 16/49) than in the control group (9%, 4/47) at posttest (*P*=.02). Because of the high attrition rate, a completers-only analysis was conducted at follow-up. The experimental group showed significant improvement in depression compared to the control group both at posttest (*P*=.01) and follow-up (*P*=.01).

**Conclusions:**

The results of this study did not show a significant effect on the reduction of depressive symptoms. However, the effect size at posttest was high, which might be an indicator of the possible effectiveness of the intervention when assessed in a larger sample and robust trial. Future research should replicate our study with adequately powered samples.

**Trial Registration:**

Dutch Trial Register: NTR2303. http://www.trialregister.nl/trialreg/admin/rctview.asp?TC=2303 (Archived by WebCite at http://www.webcitation.org/6IOxNgoDu).

## Introduction

Depressive disorders are highly prevalent [1,2] and are significantly associated with an impaired quality of life [3,4]. It is estimated that the prevalence of depression varies in different ethnic populations. For example, a European study showed that the prevalence of depressive symptoms among adult ethnic minorities was significantly higher than among native people [5]. Lower socioeconomic conditions and discrimination against ethnic minorities have been found to be important predictors for these differences. Research shows that Turkish people in the Netherlands, one of the largest ethnic minority groups in the country, have the highest 1-month prevalence of depressive and/or anxiety disorders (18.7%) in comparison with Dutch (6.6%) and Moroccan (9.8%) people [6]. Furthermore, it has been found that young women of Turkish and South-Asian descent in the Netherlands are at increased risk for committing suicide. Social oppression is perceived as one of the risk factors contributing to this higher suicidal risk [7].

Despite the fact that ethnic minorities encounter a higher risk for depression compared to the ethnic majority, they seem to receive less professional help from mental health care services than native people in Western countries [8,9]. Several reasons have been found for this lower uptake. For example, people from ethnic minorities seek mental health care at a later and more advanced stage of their mental health problems. They also have a higher chance of dropping out from therapy prematurely [10]. To lower the access threshold, it is important to apply effective recruitment strategies and to provide culturally sensitive interventions for ethnic minorities.

Psychotherapy, such as cognitive behavior therapy [11,12] and problem-solving therapy [13], has found to be effective in the treatment of adult depression, but little is known about whether this effectiveness also holds for ethnic minorities. Data are mostly obtained from studies among white, middle-income populations, leaving ethnic minorities underrepresented in clinical research [14].

However, a recent meta-analysis taking ethnic minorities into consideration showed a first indication that psychotherapy may be equally as effective in ethnic minorities as in native populations [15]. Therefore, this finding would justify strategies for lowering the access threshold to psychotherapy for ethnic minorities with depression. One such way could be the delivery of depression interventions by Internet. Because the Internet can overcome several barriers to treatment uptake, it could help in reaching out to ethnic minorities with unmet needs for treatment. It can lower the access threshold and provide anonymity and considerable flexibility in terms of time and place.

Internet interventions have proved to be effective in the treatment of depressive symptoms and the prevention of depression, as shown in a number of studies [16-18]. However, it is unclear whether this evidence for Internet-based interventions can be generalized to ethnic minority groups. So far, few studies have focused on ethnic minorities in online trials. For example, a recent Australian study showed promising results for an Internet-based cognitive behavior therapy in the treatment of depression in Chinese migrants [19]. The participants in the experimental group evaluated the Internet-based treatment as acceptable and reported significantly reduced depressive symptoms (Cohen’s *d*=0.93) up to 3 months after treatment compared to a control group.

In the Netherlands, one such successful Internet-based, guided, self-help intervention based on problem-solving therapy is Alles Onder Controle (AOC; Everything under Control). AOC has been shown to be clinically effective in the reduction of depressive symptoms with a moderate effect size (Cohen’s *d*=0.50) [20,21]. This intervention appears also to be cost-effective as shown by Warmerdam and colleagues [22]. For the purpose of this study, AOC was adapted to the specific needs of Turkish people living in the Netherlands (AOC-TR) [23]. We investigated the effectiveness of AOC-TR by means of a randomized controlled trial with a similar design as the trial of Warmerdam and colleagues [21]. We hypothesized that Turkish adult migrants in the experimental group would show a significant reduction in depressive complaints compared to those in a waitlist control group. To the best of our knowledge, this is the first study evaluating a culturally sensitive, Internet-based, self-help intervention for Turkish migrants with depressive complaints.

## Methods

### Trial Design

A two-armed randomized controlled trial was conducted to examine the effectiveness of AOC-TR, comparing the experimental group with a waiting list control group. The experimental group obtained direct access to the intervention and the waiting list control group received access after 4 months. Ethical approval was granted by an independent medical ethics committee (METc VUmc registration no: 2010/82). The trial is registered in the Dutch Trial Register (NTR2303). A detailed description of the trial design has been published earlier [23].

### Participants and Inclusion/Exclusion Criteria

Participants aged 18 years or older with depressive symptoms as measured by a Center for Epidemiologic Studies Depression Scale (CES-D) score ≥16 [24] and a Turkish background (participant or at least 1 parent was born in Turkey) were included in the trial. To be included, participants also needed to have access to a computer with Internet, have an email address, and have returned a signed informed consent form.

Exclusion took place if the participant was suicidal, which was assessed in 2 steps as part of the online screening. First, the suicide item on the Beck Depression Inventory II (BDI-II) was presented [25,26]. The BDI-II is validated among Dutch [27,28] and Turkish populations [29,30]. Second, if the response was affirmative, the suicide risk was measured with the suicidality section of the Mini-international Neuropsychiatric Interview (MINI) [31,32] in Dutch [33] or Turkish [34]. Participants with a relatively high risk were advised to contact their general practitioner or were referred to the online portal for suicide prevention [35].

### Recruitment

Recruitment took place from June 16, 2010 to March 15, 2012. Participants were recruited among the adult Turkish migrant population via several recruitment strategies. The following strategies were applied: advertisements in Dutch and Turkish national newspapers, magazines, and community websites; banners on health-related websites for migrants; and through social media. Information brochures were distributed at Turkish associations in the Netherlands, mental health care organizations, and sociocultural organizations. The recruitment took place in 2 languages, Dutch and Turkish. Facebook was the most effective recruitment strategy during the trial.

Recruitment on Facebook took place between January 1, 2011 and March 15, 2012. A personal profile and a fan page about the trial were created on Facebook, where pictures from the research website, information about the project, and status updates were shared. Facebook groups related to Turkish migrants and Turkish groups focusing on (general) health and psychology were joined. Next, random people from these groups were invited to join the fan page and friend requests were sent. A total of 584 friend requests were accepted by these invited people. Afterwards, friends of our friends list and people from the Facebook groups began to add our Facebook profile, which resulted in 3308 friends on the research profile by the end of the trial. Friends from our friends list and from the joined Facebook groups sent us messages or, if the researcher was online, chat conversations took place with them. We received or had chat conversations (about diverse topics, including application to the trial) with 348 people.

The advertisements contained a link to our research website with detailed information about the trial [36,37]. Interested parties could apply by sending an email to the researcher, who then returned a digital information brochure about the study, the informed consent form, and a unique Web link for an online screening questionnaire.

### Intervention

The original version of the self-guided, problem-solving intervention (AOC [20]) was adapted to a culturally sensitive intervention (AOC-TR) in collaboration with the Trimbos Institute (Netherlands Institute of Mental Health and Addiction). First, the intervention was translated from Dutch into Turkish, and then both versions were culturally adapted. Although there are multiple descriptions of cultural adaptation of psychotherapy to specific populations, it has been defined as the modification of intervention protocols according to the clients’ values, contexts, and worldviews [38]. Culture-specific adaptations in our intervention included several components: (1) the participants’ preferred language, (2) describing psychological problems in terms of idioms of distress (eg, using symptoms of depression instead of the term depression), (3) explicitly discussing migration and culture by using culture-specific cases and problems that are recognizable for the target group concerned, and (4) including recognizable examples of persons with similar problems (eg, a young woman who migrated 2 years ago and can’t find her way in the Netherlands). After adapting the intervention from the original Dutch version, 2 native Turkish persons evaluated the interventions both for language- and cultural-specific items in close collaboration with the first author who is a Turkish person herself. Finally, recommendations from these reviewers in terms of culture and language were incorporated in the interventions. Screenshots of the interventions are shown in Multimedia Appendices 1 and 2.

The AOC-TR consists of 5 sessions over 5 weeks. During the intervention, participants indicate what they think is important in their lives, they make a list of their problems and worries, and they categorize their problems into 3 groups: (1) unimportant problems, which are not related to what they think is important in their lives, (2) important and solvable problems, which are approached by a systematic problem-solving approach consisting of 6 steps, and (3) important but unsolvable problems, such as having lost someone through death or having a chronic general medical disease and making a plan for how to live with it. The core of the intervention is the 6-step problem-solving procedure, which teaches to use this technique during the course for several of their important and solvable problems. The idea is that by mastering this technique people will regain mastery of their problems and ultimately their lives.

The participants received feedback on their homework assignments in brief weekly emails in either Turkish or Dutch from the researcher (BÜI).

### Control Condition

The control condition was a waiting list comparator; participants in this condition did not receive access to the intervention after randomization. However, they were provided with access to the intervention 4 months after the baseline measures.

### Outcome Measures

#### Overview

Assessments took place before randomization (T0), after completing the treatment (8 weeks, T1), and 4 months after baseline (T2). All assessments were offered in the preferred language of the participant, either Dutch or Turkish.

#### Primary Outcome Measure: Depressive Symptoms

Depression severity was measured with the CES-D [24] including 20 self-rated items, each scored from 0 to 3. The Dutch [39], Turkish [40], and online [41] versions of the CES-D have been proven to have good psychometric properties in terms of validity and reliability. In the current study, the internal consistency was good (Cronbach alpha =.87 at baseline).

#### Secondary Outcome Measures

##### Anxiety

The anxiety scale of the Hospital Anxiety and Depression Scale (HADS) was used to measure symptoms of anxiety [42]. The HADS consists of an anxiety scale with a total of 7 items. Each item is scored on a 4-point Likert scale within a range of 0 to 3 (low to high). The HADS has proven to be a valid and reliable instrument in various normal and clinical Dutch [43] and Turkish samples [44]. The Cronbach alpha coefficient was .78 at baseline in the current study.

##### Somatic Symptoms

To measure somatic symptoms, the somatization subscale on the Symptom Checklist-90-Revised (SCL-90-R) was used [45]. This is a 5-point rating scale containing 12 items. Dutch [46] and Turkish translations [47] were used for this study, both having good reliability and validity. In the current study, the Cronbach alpha coefficient was .86 at baseline.

##### Quality of Life

Quality of life was measured using the EuroQol Questionnaire (EQ-5D) [48,49] in the official Dutch and Turkish translations, both of which have been validated [50,51]. The last item on the EQ-5D, the EQ visual analogue scale (EQ-VAS), was used in which the health state of the participant is measured by a thermometer-like scale from 0 (worst) to 100 (best health state).

##### Satisfaction With the Treatment

Participants were asked to define their satisfaction with each lesson by asking, “Was this lesson useful to you?” in Dutch and Turkish. The answers could be rated on a 5-point Likert scale. The score per item ranged from 1 (not at all) to 5 (very much). The Cronbach alpha coefficient was .90 at T1.

#### Additional Measures

Sociodemographic information (sex, age, country of birth of participant and participant’s parents, educational level, employment, and long-term relationship or partner status) and additional information were collected about how the participants were referred to the trial, why they chose an Internet-based intervention, and whether they use the Internet for health-related topics.

### Sample Size

The sample size was calculated on an expected difference of *d*=0.45 between the experimental and control groups. This expected difference was based on effect sizes derived from previous effect studies on Internet-guided problem-solving therapy for depression [21]. To achieve a power of 0.80 and an alpha of .05, we needed 78 participants at baseline in each condition (N=156). In keeping with our hypothesis, the primary and secondary outcomes were analyzed with a 1-tailed *t* test as in the study of Warmerdam and colleagues [21].

### Randomization

Participants were randomly assigned to the experimental or the control group after baseline assessment. The allocation schedule was generated by an independent researcher using a computerized system.

### Analyses

#### Overview

The study was carried out in accordance with the CONSORT guidelines [52]. Differences in demographic characteristics were computed with a chi-square test. For small samples, the likelihood ratio test was performed. Clinical outcomes, differences in baseline, posttest, and follow-up mean scores (at T0, T1, and T2) were analyzed with a *t* test.

#### Missing Values

Only posttreatment data were analyzed according to the intention-to-treat principle. Missing values were handled using the multiple imputation technique in SPSS Statistics version 20.0 (IBM Corp, Armonk, NY, USA). All variables (except nominal variables) were included as predictors and generated 100 imputations. Analyses were performed using pooled data.

#### Effect Sizes

For comparison of the 2 means, Cohen’s *d* was used to determine the between-group effect size at posttreatment and follow-up [53]. Cohen’s *d* was calculated as the difference between the posttest mean scores of the intervention and the control group divided by the pooled standard deviation. Effect sizes of 0.8 are assumed to be large, effect sizes of 0.5 are moderate, and effect sizes of 0.2 are assumed to be small [53].

#### Clinically Significant Change

Analyses of clinically significant change on the CES-D were conducted according to the Jacobson and Truax formula [54]. This method evaluates 2 criteria for each participant. The first is whether each participant’s CES-D score improved such that it is unlikely to be due to chance (reliable change index, RCI). The RCI is a function of a participant’s pretest and posttest scores, the standard deviation of the population before treatment, and the test-retest reliability of the measure [54,55]. A participant is considered to have experienced reliable change if his or her RCI is greater than 1.96 [56]. The second criterion evaluated for participants shown to have reliable change is whether their posttreatment symptom level places them at a score of 16 or lower on the CES-D. Clinically significant change was determined if the participant had recovered and shown reliable improvement over time.

#### Per-Protocol Analysis

Per-protocol analyses were performed for participants who completed all the measurements and all 5 lessons of the course (if randomized to the experimental condition).

## Results

### Participants

**Figure 1:**
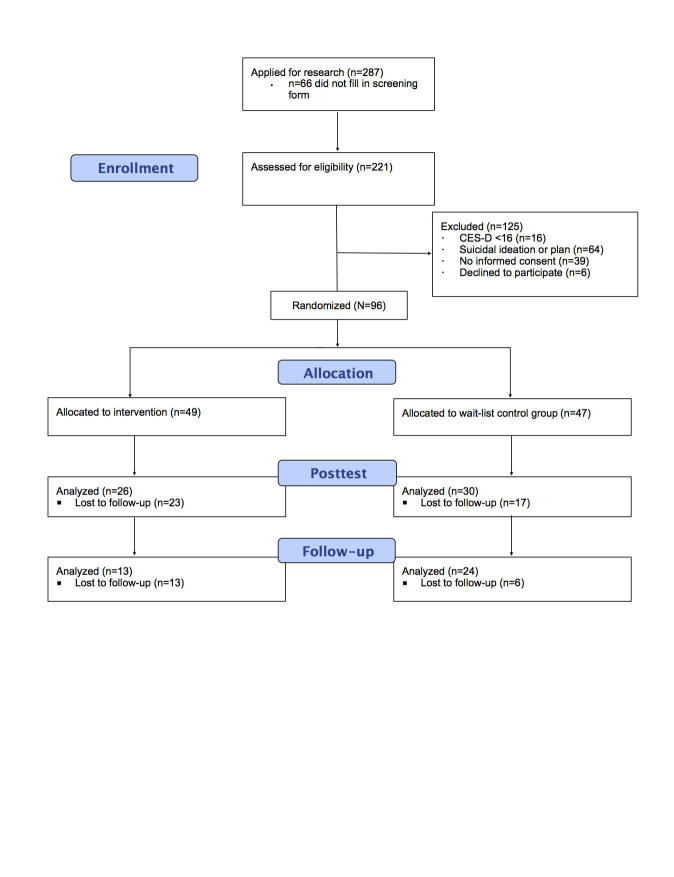
CONSORT flow diagram of the participation progress through the trial.

Figure 1 shows the flow of participants through the trial. A total of 287 individuals applied for participation. However, 66 of them did not complete the screening. The screening questionnaire was filled in by 221 individuals, of whom 125 were excluded primarily because of suicidal ideations (64/125, 51.2%). A total of 96 individuals met all inclusion criteria and were randomized to 1 of the 2 conditions.

Table 1 provides the baseline characteristics of study participants. The mean age of the participants was 35.2 years (SD 9.3) and 62% (59/96) were women. Most participants were born in Turkey (91%, 87/96) and preferred the Turkish language for study participation (89%, 85/96). More than three-quarters of the participants (78%, 75/96) were recruited through the Internet. The most important reason for choosing an Internet intervention was flexibility of use (62%, 59/96), followed by privacy and anonymity (23%, 22/96).

The mean score at baseline for all the participants on the CES-D was 29.9 (SD 9.6, range 11-52). There were no statistically significant differences between the experimental and control group at baseline on any of the demographic and secondary outcomes.

### Attrition

Of the 96 original participants, a total of 40 (42%) participants did not complete the posttest (6 weeks), and 59 participants (62%) did not complete the follow-up assessment at 4 months. Reasons for the high attrition rates are not known. There were no significant differences in attrition rates between the experimental (47%, 23/49) and the control group (36%, 17/47) at posttest (*P*=.29). However, at follow-up, the experimental group (74%, 36/49) had a higher attrition rate than the control group (49%, 23/47; *P*=.01).

### Effects of the Intervention at Posttest

#### Intention-to-Treat Analysis

Table 2 shows the outcomes for the primary (CES-D) and secondary (HADS, SCL-90, and EQ-5D) measures at posttreatment. The results show no difference between the experimental and the control group at posttest for the primary outcome assessed with the CES-D (*P*=.07; Cohen’s *d*=0.37, 95% CI –0.03 to 0.78). We did not find any significant differences between the 2 groups on the secondary outcomes.

#### Clinically Significant Change

Data on clinically significant change are shown in Table 3. In the intention-to-treat sample, the experimental group (32.9%) had significantly higher recovery rates on the CES-D than the control group (9.4%, *P*=.02) at posttest. However, no differences between the experimental and control group were found for improvement or clinically significant change.

#### Per-Protocol Analysis

The outcomes for participants who fulfilled the protocol for intervention and outcome assessments are shown in Table 4. Several significant outcomes at posttest assessments can be observed. At posttest, the experimental group showed a significantly greater improvement in depressive symptoms compared to the control group (*P*<.001) with a large effect size of *d*=1.68 (95% CI 0.69-2.67). Differences were also found in favor of the experimental group for reduction of anxiety symptoms (*P*<.001), with a large effect size of *d*=1.48 (95% CI 0.51-2.45) and also in somatization symptoms (*P*<.001), with a large effect size of *d*=1.37 (95% CI 0.41-2.33) compared to the control group.

#### Completers-Only Analysis

Table 5 shows the outcomes for responding participants at posttest assessments in comparison with the control condition. Results show a significantly greater improvement in depressive symptoms in the experimental group than the control group at posttest (*P*<.001), with a large effect size of *d*=0.72 (95% CI 0.18-1.26). We did not find any significant differences between the 2 groups on the secondary outcomes at posttest.

#### Sessions Attended and Satisfaction With Treatment

A total of 18 of 49 (37%) participants who were assigned to the experimental group did not start the treatment. Of those who started, 12 of 49 (26%) participants completed 1 to 2 lessons, 9 of 49 (18%) participants completed 3 or 4 lessons, and 10 of 49 (20%) participants completed all 5 lessons. Participants who completed the treatment expressed moderate satisfaction (total score mean 2.75, SD 0.96) with the intervention.

### Effects of the Intervention at Follow-Up: Based on Completers-Only Sample

Because of high attrition, we conducted completers-only analysis for follow-up results at 4 months after the start of the intervention. Results are shown in Tables 3-5. As is shown, the experimental group did significantly better on the primary and secondary outcomes analyses (clinically significant change, per-protocol analysis, and completers-only analysis).

**Table 1:** Demographic characteristics and baseline test scores at T0 (N=96).

Demographic characteristics and baseline tests		Total (N=96)	Experimental group (n=49)	Control group (n=47)	*P* value^a^
Age (years), mean (SD)		35.2 (9.3)	34.9 (8.9)	35.6 (9.8)	.72
Gender (female), % (n)		62 (59)	65 (32)	57 (27)	.43
Born in Turkey, % (n)		91 (87)	92 (45)	89 (42)	.68
Long-term relationship, % (n)		64 (61)	71 (35)	55 (26)	.10
**Educational level, % (n)** ^b^					
	Low	27 (26)	35 (17)	19 (9)	
	Middle	41 (39)	31 (15)	51 (24)	
	High	32 (31)	35 (17)	30 (14)	.09
Preference for Turkish language, % (n)		89 (85)	88 (43)	89 (42)	.81
**Recruitment channel, % (n)**					
	Internet	78 (75)	78 (38)	79 (37)	
	Internet through Facebook	99 (74)	100 (38)	97 (36)	
	Newspaper	1 (1)	0 (0)	2 (1)	
	Magazine	1 (1)	2 (1)	0 (0)	
	Friends or family	6 (6)	10 (5)	2 (1)	
	Other	14 (13)	10 (5)	17 (8)	.89
Employed, % (n)		52 (49)	45 (22)	57 (27)	.22
**Reason for choosing Internet intervention, % (n)**					
	Privacy/anonymity	23 (22)	22 (11)	23 (11)	
	Flexibility	62 (59)	51 (25)	72 (34)	
	Other	16 (15)	27 (13)	4 (2)	.01
**Use of the Internet for health information, % (n)**					
	Physical complaints	7 (7)	8 (4)	6 (3)	
	Psychological complaints	10 (10)	8 (4)	13 (6)	
	Physical and psychological complaints	67 (64)	71 (35)	62 (29)	
	None	16 (15)	12 (6)	19 (9)	.65
**Test outcomes, mean (SD)**					
	Depression (CES-D)	29.9 (9.6)	29.6 (9.2)	30.1 (10.1)	.79
	Anxiety (HADS)	13.0 (4.1)	13.3 (4.3)	12.7 (3.9)	.52
	Somatization (SCL-90)	30.0 (8.6)	31.0 (9.2)	29.0 (8.0)	.25
	Quality of life (EQ-VAS)^b^	60.4 (21.2)	57.7 (21.5)	63.14 (20.8)	.27

^a^CES-D was analyzed with a 1-tailed *t* test. The other tests were analyzed with a 2-tailed *t* test.

^b^Total: N=74; experimental group: n=38; control group: n=36. This item was the last of the assessment, which was not filled in by every participant.

**Table 2:** Study outcomes at posttest including posttest between-group effect size (Cohen’s *d*): intention-to-treat analysis.

Outcome and group			Pretest		Posttest		*P* value	Mean difference	Cohen’s *d* (95% CI)
			n	Mean	n	Mean			
**Primary outcome**									
	**Depression (CES-D)**								
		Experimental group	49	29.6	49	23.0			
		Control group	47	30.1	47	27.2	.07	–4.25	0.37 (–0.03, 0.78)
**Secondary outcomes**									
	**Anxiety (HADS)**								
		Experimental group	49	13.3	49	11.0			
		Control group	47	12.7	47	11.7	.23	–0.76	0.25 (–0.16, 0.65)
	**Somatization (SCL-90)**								
		Experimental group	49	31.0	49	28.1			
		Control group	47	29.0	47	28.0	.48	0.13	0.15 (–0.26, 0.55)
	**Quality of life (EQ-VAS)**								
		Experimental group	38	57.7	38	65.4			
		Control group	36	63.1	36	65.7	.48	–0.28	0.15 (–0.26, 0.55)

**Table 3:** Clinically significant change analyses of depression tested by the CES-D.

Clinically significant change analyses		Posttest, n (%)		*P* value	Follow-up, n (%)		*P* value
		Experimental group	Control group		Experimental group	Control group	
**Intention-to-treat analysis**							
	Recovery	15.6 (32.9)	4.3 (9.4)	.02			
	Improvement	18.5 (37.8)	9.4 (20.0)	.07^b^			
	Clinically significant change	11.8 (24.9)	2.6 (5.7)	.05			
**Completers only**							
	Recovery	10 (38.5)	1 (3.3)	<.001	6 (46.2)	3 (12.5)	.01
	Improvement	12 (46.2)	4 (13.3)	.01	7 (53.8)	4 (16.7)	.01
	Clinically significant change	6 (23.1)	1 (3.3)	.01	5 (38.5)	2 (8.3)	.01

^a^Recovery was defined as having a CES-D score below 16. Improvement was defined as having a reliable change if the individual RCI is greater than 1.96. Clinically significant change was determined if both recovery and improvement took place.

^b^For this analysis, the *P* value of the chi-square analysis is provided.

**Table 4:** Study outcomes of participants at posttest and follow-up including between-group effect size (Cohen’s *d*): per protocol (n=30).

Per protocol			Posttest				4-month follow-up			
			n	Mean (SD)	*P*	Cohen’s *d* (95% CI)	n	Mean (SD)	*P*	Cohen’s *d* (95% CI)
**Primary outcome**										
	**Depression (CES-D)**									
		Experimental group	6	15.3 (9.9)			6	19.0 (13.9)		
		Control group	24	29.5 (8.8)	<.001	1.68 (0.69, 2.67)	24	30.1 (11.3)	.02	1.13 (0.19, 2.07)
**Secondary outcomes**										
	**Anxiety (HADS)**									
		Experimental group	6	7.3 (2.9)			6	7.8 (4.9)		
		Control group	24	12.4 (3.5)	<.001	1.48 (0.51, 2.45)	24	12.1 (3.7)	.01	1.26 (0.31, 2.21)
	**Somatization (SCL-90)**									
		Experimental group	6	18.8 (6.2)			6	19.2 (6.9)		
		Control group	24	28.5 (8.1)	.001	0.37 (0.41, 2.33)	24	28.5 (8.5)	.01	1.27 (0.32, 2.22)
	**Quality of life (EQ-VAS)**									
		Experimental group	5	79.4 (28.9)			5	82.3 (23.0)		
		Control group	20	63.6 (21.7)	.07	0.95 (–0.06, 1.97)	20	66.4 (23.2)	.11	0.83 (–0.18, 1.84)

**Table 5:** Study outcomes of participants at posttest and follow-up including follow-up between-group effect size (Cohen’s *d*): completers only (n=56).

Completers only			Posttest				4-month follow-up			
			n	Mean (SD)	*P*	Cohen’s *d* (95% CI)	n	Mean (SD)	*P*	Cohen’s *d* (95% CI)
**Primary outcome**										
	**Depression (CES-D)**									
		Experimental group	26	21.38 (10.5)			13	21.23 (10.79)		
		Control group	30	28.27 (8.71)	.01	0.72 (0.17, 1.26)	24	30.08 (11.27)	.01	0.94 (0.23, 1.65)
**Secondary outcomes**										
	**Anxiety (HADS)**									
		Experimental group	26	10.54 (4.00)			13	9.69 (4.92)		
		Control group	30	11.87 (3.76)	.10	0.45 (–0.08, 0.98)	24	12.08 (3.67)	.05	0.69 (0.00, 1.39)
	**Somatization (SCL-90)**									
		Experimental group	26	26.00 (10.02)			13	25.31 (9.78)		
		Control group	30	27.70 (7.76)	.24	0.32 (–0.21, 0.85)	24	28.54 (8.52)	.15	0.51 (–0.18, 1.19)
	**Quality of life (EQ-VAS)**									
		Experimental group	19	70.95 (19.52)			10	78.80 (22.50)		
		Control group	23	64.17 (21.54)	.15	0.46 (–0.16, 1.07)	19	66.42 (23.19)	.13	0.61 (–0.17, 1.39)

## Discussion

### Principal Results

Our results show no significant difference in improvement of depressive complaints in the experimental group compared to the control group on intention-to-treat analysis at posttest. This may be because our study was underpowered [57] (see also the limitations section). However, we found an effect size for the primary outcome (depression) of *d*=1.68 at posttest and *d*=1.13 at follow-up, which is an indication that the intervention could be effective with a sample size indicative of sufficient power. We did not find any differences on secondary outcomes. Recovery occurred significantly more often in the experimental group than in the control group at posttest (*P*=.01) but clinically significant change was not (*P*=.09). Completers-only analyses showed that the results of the analysis for the primary outcome differed from the imputation data, suggesting that the analysis was influenced by data imputation.

### Comparison With Previous Work

Our results did not support the effectiveness of the Internet-based, guided, self-help intervention, in contrast to the original version (AOC) [20]. As mentioned before, AOC was previously shown to be clinically effective in the general Dutch population in the reduction of depressive symptoms with a moderate effect size (Cohen’s *d*=0.50). However, the effect size at posttest in the current study was high, which might be an indicator of the possible effectiveness of the intervention for Turkish migrants when assessed in a larger sample.

The inclusion of ethnic minorities in clinical research has been a challenge for many years. Ethnic minorities are generally underrepresented in scientific and clinical analyses and are known to be a hard-to-reach population for research purposes [8,58]. Although previous research shows that ethnic minorities may have participated in Internet interventions (eg, [59]), randomized controlled trials on the effectiveness of psychotherapy for common mental disorders, such as depression, are still sparse. Ethnic minorities are underrepresented in mental health research and literature about ethnic differences in this field is very small [8]. In our recent meta-analysis, we found only 56 randomized controlled trials on the psychological treatment of depression in adults reporting the proportion of participants from ethnic minorities, of which none of them made distinct comparisons between ethnic populations [15].

It is generally believed that ethnic minorities are less willing to participate in clinical research; however, very small differences between ethnic minorities and majorities are found in the willingness to participate in health research [60]. Other factors, such as higher costs associated with the recruitment, the exclusion criterion of insufficient ability to speak the second (native) language, the shortage of ethnic minority coordinators in trials, and stereotypes and myths, are considered to be important barriers for their participation [61].

Our study shows that recruitment of ethnic minorities is possible when appropriate recruitment strategies are applied. For example, almost 80% of our participants were recruited through the Internet (primarily on Facebook). Traditional media, such as advertisements in newspapers or banners on websites, appear not to be successful recruitment strategies for this target group, although many studies have applied this strategy successfully for recruiting participants for randomized controlled trials and studies in routine practice among the general population [21,62]. Flexibility and privacy of the Internet were the main reasons for respondents to agree to participate. The use of social media in research is a relatively new development, and may potentially prove more effective for recruiting ethnic minorities in research trials. The contact through social media and the visibility of the researcher seemed to lower the threshold for participation in research and for help seeking. Although we did not find significant results from the intervention, the current trial shows that the Internet (1) is an effective way to reach hard-to-reach populations, (2) lowers the threshold to get in contact with a professional, (3) can be an effective recruitment strategy for clinical trials, and (4) is potentially an effective way to deliver cognitive behavioral therapy for ethnic minorities.

Furthermore, our participants consisted primarily of first-generation migrants who had a preference for the Turkish (native) language. Offering the intervention and assessment measures in 2 languages may have been another successful strategy to lower the threshold for study participation. Generally, participants are only included in intervention studies when they can read and speak the language of the country they live in [61].

Another argument for the low-access threshold of our intervention might be found in the large number of applicants with suicidal ideation (30%, 64/221). We had to exclude these applicants (51.2% of excluded group) because they are a high-risk group not suited to our guided self-help intervention. In keeping with the protocol, we referred these individuals to their primary care physician or to the online portal for suicide prevention [35]. These applicants were primarily women (59.4%) with a mean age of 33.5 years (range 18-53).This is a rather high number when compared with those excluded because of suicidal ideation among primarily native population studies [63,64]. The number also appears high when compared to prevalence rates at the population level. For example, in the Netherlands, 8.3% of the Dutch population have ever had suicidal ideation in their lifetime and 2.2% have attempted suicide [65]. As mentioned in the introduction, young Turkish women are at increased risk of committing suicide [7]. It seems that our study has reached a large number of this group. Future Internet research could focus more specifically on this high-risk group. These individuals experience a high burden of disease and unmet needs, but appear to be reachable by Internet. Thus, the delivery of psychotherapy through the Internet appears to be a promising way to target hard-to-reach ethnic minority groups.

### Limitations

This study has several limitations. First, the attrition ratio was high at posttest. We compensated for this high attrition by means of multiple imputation. Attrition was even higher at follow-up; therefore, we decided not to apply multiple imputation for this time point. Instead, we conducted completers-only analyses. High attrition remains a common problem in Internet interventions, with rates of up to 50% [66,67]. Analyses showed no differences in study attrition rates at posttest between the experimental group and control group. However, at follow-up, the experimental group had a higher attrition rate than the control group. Reminders in the form of emails were sent (maximum 5 times per assessment), but this did not result in a low attrition rate for study dropout. Reasons for this high attrition rate are not known; as a result, we can only guess why this happened. It is possible that participants in the experimental group stopped with the trial after finishing the intervention. Filling in the posttreatment questionnaires might not have been regarded as an obligatory part of the trial. Furthermore, the control group was waiting to receive access to the intervention, they could have perceived filling in the questionnaires as an obligation to partake in the intervention.

Second, although we reached a relatively large number of Turkish migrants, recruitment and inclusion were challenging and complex. One of the challenges was to find an appropriate recruitment strategy. In the end, we did find one (ie, Facebook), but we had only limited time left for recruitment because of the overall period available for this study. This could be one of the main reasons for not having been able to obtain the required sample size. Another problem was that we had to exclude most of the eligible participants for reasons such as high suicidality risk. Thus, we did not achieve the target sample size (N=200) during the study period, which may have resulted in an underpowered study. In turn, this may have been the reason that significant effects were not detected and it limited the generalizability of our results.

Third, our target population focused on the online population because participants were required to have access to the Internet and an email address to be included in our trial. Moreover, it is known that almost 80% of the Turkish population in the Netherlands has Internet access [68]. However, our population may have differed from most Turkish people in another way. When we look at the demographic characteristics of our sample, we notice that younger women (mean 35.2 years) with a middle to higher educational level (70%) took part. This is a higher proportion than the Turkish population in the Netherlands, of which 30.1% had at least a middle educational level [69]. Our sample conforms to the sample characteristics of nonmigrant populations in similar trials, in which women (aged 35-55 years) with higher educational levels have taken part [20,21].

Furthermore, although participants were required to have access to a computer with Internet and have an email address, we did not assess the reading and Internet comfort level of the participants. Given that most of our respondents were recruited through Facebook, we assume that respondents at least were able to understand our intervention and questionnaires. Next, respondents could choose the language in which they wanted to follow the intervention and answer the questionnaires (Turkish or Dutch).

Finally, we used only self-report assessments to measure the severity of depressive symptoms in participants. We used self-report on purpose because we wanted to keep the access barrier for study participation as low as possible. Diagnostic interviews are an extra burden for participants and it is not yet possible to conduct them through the Internet. Therefore, our study lacks a diagnosis of depression in the study participants. Research has shown that online self-report questionnaires have good validity (eg, [41]) and yield scores equivalent to paper-and-pencil questionnaires (eg, [70]).

### Future Research and Implications

The results of this study have promising implications for the clinical field. Our study is one of the first to assess the clinical effectiveness of guided self-help interventions by Internet for Turkish migrants with depressive complaints. In addition, the guided self-help intervention for Turkish migrants in the Netherlands could also be suitable for Turkish populations in other EU countries or in Turkey itself, where guided self-help is not yet common practice. It may be a welcome intervention both for clinicians and for minorities because there is a lack of evidence-based culturally sensitive psychotherapy for ethnic minorities and there is a high threshold to these services.

Future research should replicate our findings with adequately powered samples for posttest and follow-up measurement to assess the clinical effectiveness in a robust manner. Future research should focus on monitoring participants who drop out prematurely from the study at follow-ups to evaluate the reasons for withdrawal. It is also important to evaluate the impact of culturally sensitive components in Internet interventions for ethnic minority populations with depression.

### Conclusions

The results of this study did not show a significant effect on the reduction of depressive symptoms. However, the effect size at posttest was high, which might be an indicator of the possible effectiveness of the intervention when assessed in a larger sample and robust trial. Future research should replicate our study with adequately powered samples.

## Multimedia Appendix 1

Screenshot of the Turkish version of AOC-TR.

## Multimedia Appendix 2

Screenshot of the Dutch version of AOC-TR.

## Multimedia Appendix 3

CONSORT-EHEALTH checklist V1.6.2 [71].

## Abbreviations

AOC: Alles Onder Controle (Everything under Control)
AOC-TR: Alles Onder Controle TR (Turkish adapted version)
BDI-II: Beck Depression Inventory II
CES-D: Center for Epidemiologic Studies Depression Scale
EQ-5D: EuroQol Questionnaire 5D
EQ-VAS: EuroQol visual analogue scale
MINI: Mini-international Neuropsychiatric Interview
NTR: Dutch Trial Register
RCI: reliable change index
SCL-90-R: Symptom Checklist-90-Revised.
