# Supplementary figures and images for: Internet-Based, Culturally Sensitive, Problem-Solving Therapy for Turkish Migrants With Depression: Randomized Controlled Trial
Source: J Med Internet Res. 2013 Oct 11;15(10):e227. doi: 10.2196/jmir.2853 (PMC3849840; doi:10.2196/jmir.2853)

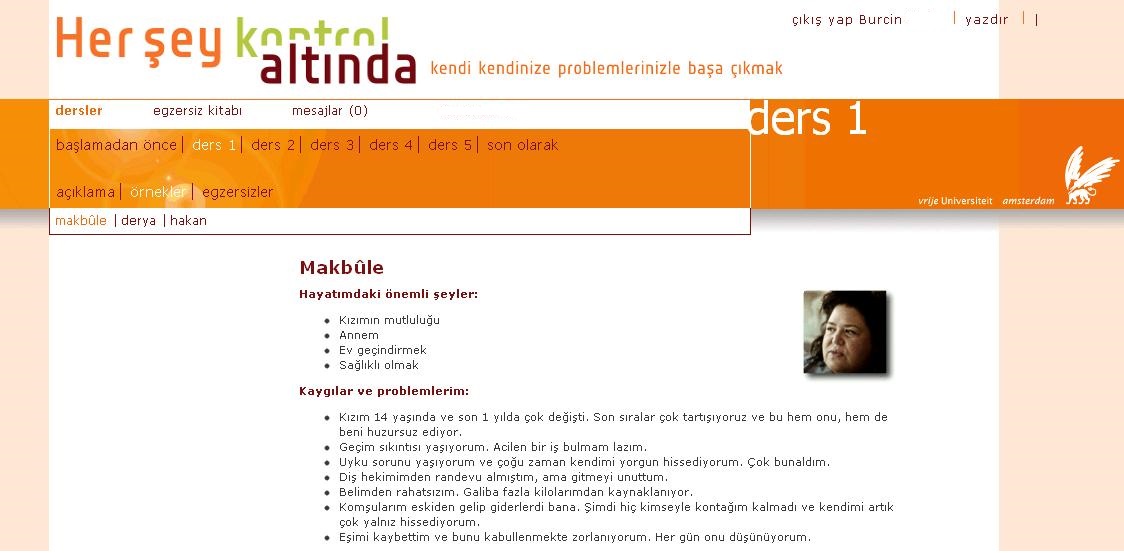

Supplement: Supplementary file 1 [file jmir_v15i10e227_app1.JPG]

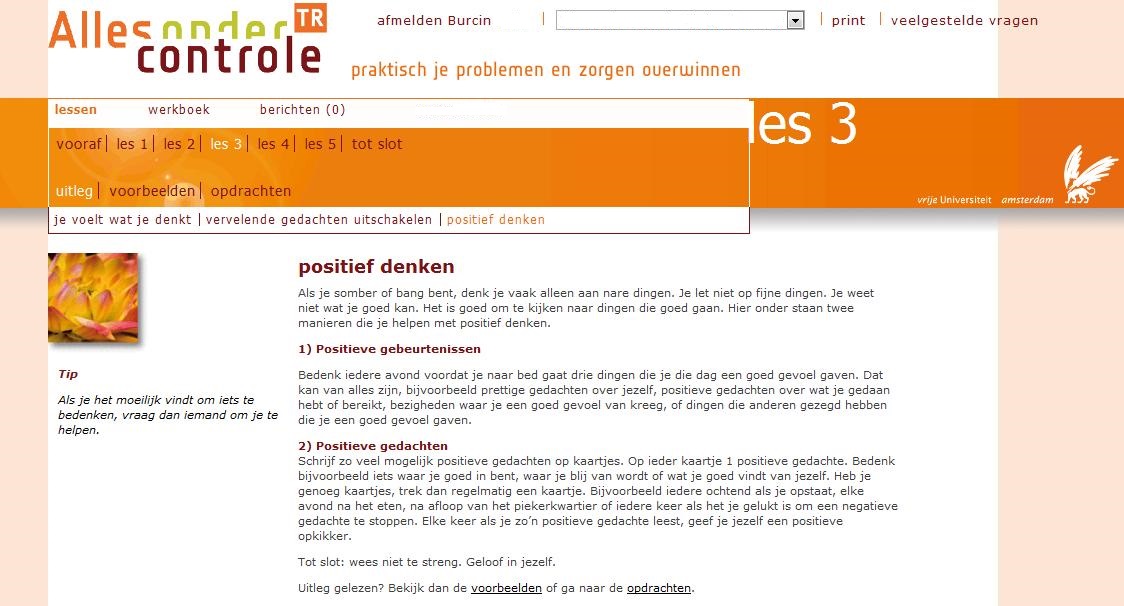

Supplement: Supplementary file 2 [file jmir_v15i10e227_app2.JPG]
