# Supplementary material for: Internet-Based, Culturally Sensitive, Problem-Solving Therapy for Turkish Migrants With Depression: Randomized Controlled Trial
Source: J Med Internet Res. 2013 Oct 11;15(10):e227. doi: 10.2196/jmir.2853 (PMC3849840; doi:10.2196/jmir.2853)
Supplement: Supplementary file 3 [file jmir_v15i10e227_app3.pdf]

CONSORT-EHEALTH Checklist V1.6.2 Report

(based on CONSORT-EHEALTH V1.6), available at [http://tinyurl.com/consort-ehealth-v1-6].

2853

Date completed

7/26/2013 11:50:16

by

Burçin Ünlü Ince

Internet-based, culturally sensitive problem solving therapy for Turkish migrants with depression: results from a randomized controlled trial

TITLE

1a-i) Identify the mode of delivery in the title

"Internet-based"

1a-ii) Non-web-based components or important co-interventions in title

No.

1a-iii) Primary condition or target group in the title

"Turkish migrants"

ABSTRACT

1b-i) Key features/functionalities/components of the intervention and comparator in the METHODS section of the ABSTRACT

"A two-armed randomized controlled trial"

1b-ii) Level of human involvement in the METHODS section of the ABSTRACT

"guided self-help"

1b-iii) Open vs. closed, web-based (self-assessment) vs. face-to-face assessments in the METHODS section of the ABSTRACT

"Participants were assessed online"

1b-iv) RESULTS section in abstract must contain use data

"Turkish adults (n=96) with depressive symptoms were randomized to the experimental group (49) or to a wait-list control group (47). High attrition rates were found among the 96 participants of which 41.7% did not complete the post-test (6 weeks) and 61.5% participants did not complete the follow-up assessment at 4 months. No significant difference between the experimental group and the control group was found for depression at post-test. However, there was a trend towards significance (P = .07) with a moderate effect size of d = .37. Recovery occurred significantly more often in the experimental group (32.9%) than in the control group (9.4%) at post-test (P =.01). "

1b-v) CONCLUSIONS/DISCUSSION in abstract for negative trials

"No significant difference between the experimental group and the control group was found for depression at post-test. However, there was a trend towards significance (P = .07) with a moderate effect size of d = .37."

INTRODUCTION

2a-i) Problem and the type of system/solution

"Turkish migrants living in the Netherlands have a high prevalence of depressive disorders but experience considerable obstacles to accessing professional help."

2a-ii) Scientific background, rationale: What is known about the (type of) system

Providing easily accessible Internet treatments may help to overcome thebarriers. In the Netherlands, one such successful Internet-based, guided self-help intervention based on problem-solving therapy is “Alles Onder Controle” (AOC) [Everything under Control]. AOC has been shown to be clinically effective in the reduction of depressive symptoms with a moderate effect size (Cohen’s d =.50). This intervention appears also to be cost-effective as shown by Warmerdam and colleagues. For the purpose of this study AOC was adapted to the specific needs of Turkish people living in the Netherlands (AOC-TR).

METHODS

3a) CONSORT: Description of trial design (such as parallel, factorial) including allocation ratio

"We hypothesized that Turkish adult migrants in the experimental group would show a significant reduction in depressive complaints compared to those in a waitlist-control group."

3b) CONSORT: Important changes to methods after trial commencement (such as eligibility criteria), with reasons

None.

3b-i) Bug fixes, Downtimes, Content Changes

None.

4a) CONSORT: Eligibility criteria for participants

"Participants aged 18 years or older, with depressive symptoms (measured by the Center for Epidemiologic Depression Scale, CES-D score ≥ 16, CES-D) [24] and a Turkish background (participant or at least one parent was born in Turkey) were included in the trial. To be included, participants also needed to have access to a PC with Internet, an e-mail address and have returned a signed informed consent form.
Exclusion took place if the participant was suicidal, which was assessed in two steps as part of the online screening. "

4a-i) Computer / Internet literacy

"To be included, participants also needed to have access to a PC with Internet, an e-mail address and have returned a signed informed consent form."

4a-ii) Open vs. closed, web-based vs. face-to-face assessments:

"Recruitment took place from June 16, 2010 to March 15, 2012. Participants were recruited among the adult Turkish migrant population via several recruitment strategies. The following strategies were applied: advertisements in Dutch and Turkish national newspapers, magazines, community websites, banners on health related websites for migrants, and via social media. Information brochures were distributed at Turkish associations in the Netherlands, mental health care organizations and socio-cultural organizations. The recruitment took place in two languages, Dutch and Turkish." All assessments were offered online in the preferred language of the participant, either Dutch or Turkish.

4a-iii) Information giving during recruitment

During recruitment: "the advertisements contained a link to our research website with detailed information about the trial [36;37]. Interested parties could apply by sending an e-mail to the researcher, who then returned a digital information brochure about the study, the informed consent form and a unique web-link for an online screening questionnaire."

4b) CONSORT: Settings and locations where the data were collected

"The following strategies were applied: advertisements in Dutch and Turkish national newspapers, magazines, community websites, banners on health related websites for migrants, and via social media. Information brochures were distributed at Turkish associations in the Netherlands, mental health care organizations and socio-cultural organizations."

4b-i) Report if outcomes were (self-)assessed through online questionnaires

"All assessments were offered online in the preferred language of the participant, either Dutch or Turkish."

4b-ii) Report how institutional affiliations are displayed

5) CONSORT: Describe the interventions for each group with sufficient details to allow replication, including how and when they were actually administered

5-i) Mention names, credential, affiliations of the developers, sponsors, and owners

"Conflicts of Interest: None declared."

5-ii) Describe the history/development process

"The original version of the self-guided problem solving intervention Alles Onder Controle was adapted to a culturally sensitive intervention (AOC-TR) in collaboration with the Trimbos Institute (Netherlands Institute of Mental Health and Addiction). First the intervention was translated from Dutch into Turkish. Then both versions were culturally adapted in terms of: 1) description of psychological problems as idioms of distress; 2) the use of culture-specific cases and problems that are recognizable for the target group concerned; and 3) culture-specific examples of persons with similar problems. AOC-TR consists of five sessions over five weeks. The participants received feedback on their homework assignments in brief weekly e-mails in either Turkish or Dutch from the researcher (B.Ü.I.). More detailed information about the intervention can be found in the protocol paper."

5-iii) Revisions and updating

5-iv) Quality assurance methods

5-v) Ensure replicability by publishing the source code, and/or providing screenshots/screen-capture video, and/or providing flowcharts of the algorithms used

5-vi) Digital preservation

"Alles Onder Controle TR. http://www.allesondercontroletr.net Archived at: http://www.webcitation.org/6IOaDsz6j: 2011."

"Her Şey Kontrol Altında. http://www.herseykontrolaltinda.net Archived at: http://www.webcitation.org/6IOaWw88g: 2011."

5-vii) Access

"The experimental group obtained direct access to the intervention, and the waiting-list control group received access after 4 months."

5-viii) Mode of delivery, features/functionalities/components of the intervention and comparator, and the theoretical framework

"The original version of the self-guided problem solving intervention Alles Onder Controle was adapted to a culturally sensitive intervention (AOC-TR) in collaboration with the Trimbos Institute (Netherlands Institute of Mental Health and Addiction). First the intervention was translated from Dutch into Turkish. Then both versions were culturally adapted in terms of: 1) description of psychological problems as idioms of distress; 2) the use of culture-specific cases and problems that are recognizable for the target group concerned; and 3) culture-specific examples of persons with similar problems. AOC-TR consists of five sessions over five weeks. The participants received feedback on their homework assignments in brief weekly e-mails in either Turkish or Dutch from the researcher (B.Ü.I.). More detailed information about the intervention can be found in the protocol paper."

5-ix) Describe use parameters

5-x) Clarify the level of human involvement

5-xi) Report any prompts/reminders used

The participants received feedback on their homework assignments in brief weekly e-mails in either Turkish or Dutch from the researcher.

5-xii) Describe any co-interventions (incl. training/support)

None.

6a) CONSORT: Completely defined pre-specified primary and secondary outcome measures, including how and when they were assessed

The primary outcome measure was the severity of depressive symptoms (CES-D); secondary outcome measures were somatic symptoms (SCL-90), anxiety (HADS), quality of life (EQ-5D) and satisfaction with the treatment. Participants were assessed online at baseline, post-test (6 weeks), and 4 months after baseline.

6a-i) Online questionnaires: describe if they were validated for online use and apply CHERRIES items to describe how the questionnaires were designed/deployed

6a-ii) Describe whether and how “use” (including intensity of use/dosage) was defined/measured/monitored

6a-iii) Describe whether, how, and when qualitative feedback from participants was obtained

6b) CONSORT: Any changes to trial outcomes after the trial commenced, with reasons

None.

7a) CONSORT: How sample size was determined

7a-i) Describe whether and how expected attrition was taken into account when calculating the sample size

7b) CONSORT: When applicable, explanation of any interim analyses and stopping guidelines

None.

8a) CONSORT: Method used to generate the random allocation sequence

"Participants were randomly assigned to the experimental or the control group after baseline assessment. The allocation schedule was generated by an independent researcher using a computerized system."

8b) CONSORT: Type of randomisation; details of any restriction (such as blocking and block size)

None.

9) CONSORT: Mechanism used to implement the random allocation sequence (such as sequentially numbered containers), describing any steps taken to conceal the sequence until interventions were assigned

Participants were randomly assigned to the experimental or the control group after baseline assessment. The allocation schedule was generated by an independent researcher using a computerized system.

10) CONSORT: Who generated the random allocation sequence, who enrolled participants, and who assigned participants to interventions

Participants were randomly assigned to the experimental or the control group after baseline assessment. The allocation schedule was generated by an independent researcher using a computerized system.

11a) CONSORT: Blinding - If done, who was blinded after assignment to interventions (for example, participants, care providers, those assessing outcomes) and how

11a-i) Specify who was blinded, and who wasn't
None.
11a-ii) Discuss e.g., whether participants knew which intervention was the "intervention of interest" and which one was the "comparator"

11b) CONSORT: If relevant, description of the similarity of interventions
Wating-list control group.
12a) CONSORT: Statistical methods used to compare groups for primary and secondary outcomes
The study was carried out in accordance with the CONSORT guidelines. Differences in demographic characteristics were computed with a Chi-square test. Clinical outcomes, differences in baseline, post-test and follow-up mean scores (at T0 T1 and T2) were analysed with a T-test.
12a-i) Imputation techniques to deal with attrition / missing values
Only post-treatment data were analysed according to the intention-to-treat principle. Missing values were handled using the multiple imputation (MI) technique in SPSS Statistics, version 20.0 [52]. All variables (except nominal variables) were included as predictors and generated 100 imputations. Analyses were performed using pooled data.
12b) CONSORT: Methods for additional analyses, such as subgroup analyses and adjusted analyses
Clinically significant change and per protocol analysis.

RESULTS

13a) CONSORT: For each group, the numbers of participants who were randomly assigned, received intended treatment, and were analysed for the primary outcome
"Turkish adults (n=96) with depressive symptoms were randomized to the experimental group (49) or to a wait-list control group."
13b) CONSORT: For each group, losses and exclusions after randomisation, together with reasons
Please see flow-diagram.
13b-i) Attrition diagram

14a) CONSORT: Dates defining the periods of recruitment and follow-up
"Participants were assessed online at baseline, post-test (6 weeks), and 4 months after baseline."
14a-i) Indicate if critical "secular events" fell into the study period

14b) CONSORT: Why the trial ended or was stopped (early)
"Although we reached a relatively large number of Turkish migrants, recruitment and inclusion were challenging and complex. One of the challenges was to find an appropriate recruitment strategy. In the end we did find one (i.e. Facebook) but we had only limited time left for recruitment due to the overall period available for this study."
15) CONSORT: A table showing baseline demographic and clinical characteristics for each group
Table 1.
15-i) Report demographics associated with digital divide issues
Table 1.
16a) CONSORT: For each group, number of participants (denominator) included in each analysis and whether the analysis was by original assigned groups
16-i) Report multiple "denominators" and provide definitions
Please see tables.
16-ii) Primary analysis should be intent-to-treat

17a) CONSORT: For each primary and secondary outcome, results for each group, and the estimated effect size and its precision (such as 95% confidence interval)
Yes.
17a-i) Presentation of process outcomes such as metrics of use and intensity of use

17b) CONSORT: For binary outcomes, presentation of both absolute and relative effect sizes is recommended
None.
18) CONSORT: Results of any other analyses performed, including subgroup analyses and adjusted analyses, distinguishing pre-specified from exploratory
Clinically significant change and per protocol analyses.
18-i) Subgroup analysis of comparing only users

19) CONSORT: All important harms or unintended effects in each group
None.
19-i) Include privacy breaches, technical problems

19-ii) Include qualitative feedback from participants or observations from staff/researchers

DISCUSSION

20) CONSORT: Trial limitations, addressing sources of potential bias, imprecision, multiplicity of analyses
20-i) Typical limitations in ehealth trials
High attrition rates and the use of only self-report assessments are discussed.
21) CONSORT: Generalisability (external validity, applicability) of the trial findings
21-i) Generalizability to other populations
Yes.
21-ii) Discuss if there were elements in the RCT that would be different in a routine application setting

22) CONSORT: Interpretation consistent with results, balancing benefits and harms, and considering other relevant evidence
22-i) Restate study questions and summarize the answers suggested by the data, starting with primary outcomes and process outcomes (use)
Yes
22-ii) Highlight unanswered new questions, suggest future research

Other information

23) CONSORT: Registration number and name of trial registry

Dutch Trial Register: NTR2303. http://www.trialregister.nl/trialreg/admin/rctview.asp?TC=2303 (Archived by WebCite at http://www.webcitation.org/6IOxNgoDu)

24) CONSORT: Where the full trial protocol can be accessed, if available

Ünlü B, Riper H, Van Straten A, Cuijpers P: Guided self-help on the internet for turkish migrants with depression: the design of a randomized controlled trial. Trials 2010;11:101. PMID: 21047442

25) CONSORT: Sources of funding and other support (such as supply of drugs), role of funders

This study is supported by the VU University and the Trimbos-institute, the Netherlands Institute of Mental Health and Addiction.

X26-i) Comment on ethics committee approval

x26-ii) Outline informed consent procedures

X26-iii) Safety and security procedures

X27-i) State the relation of the study team towards the system being evaluated

No conflicts of interest.
